# Review of Hypoparathyroidism

**DOI:** 10.3389/fendo.2016.00172

**Published:** 2017-01-16

**Authors:** Ejigayehu G. Abate, Bart L. Clarke

**Affiliations:** ^1^Division of Endocrinology and Metabolism, Mayo Clinic, Jacksonville, FL, USA; ^2^Division of Endocrinology, Diabetes, Metabolism, and Nutrition, Mayo Clinic, Rochester, MN, USA

**Keywords:** hypoparathyroidism, hypocalcemia, parathyroid hormone, PTH (1-84), natpara

## Abstract

Hypoparathyroidism is a rare endocrine disorder in which parathyroid hormone (PTH) production is abnormally low or absent, resulting in low serum calcium and increased serum phosphorus. The most common cause of hypoparathyroidism is parathyroid gland injury or inadvertent removal during thyroid surgery. Current treatments include supplementation with calcium and active vitamin D, with goal albumin-corrected serum calcium level in the low-normal range of 8–9 mg/dl. Complications of the disease include renal dysfunction, nephrocalcinosis, kidney stones, extracellular calcifications of the basal ganglia, and posterior subcapsular cataracts, as well as low bone turnover and increased bone density. Until January 2015, hypoparathyroidism was the only classic endocrine disease without an available hormone replacement. Recombinant human PTH 1-84, full-length PTH, is now available for a selected group of patients with the disease who are not well controlled on the current standard therapy of calcium and active vitamin D. In addition, the role of PTH replacement on quality of life, intracerebral calcifications, cataracts, improving bone turnover, and reduction of renal complications of the disease remains to be further investigated.

## Introduction

The mechanism of action of parathyroid hormone (PTH) was first described by Collip et al. in 1912 (1, 2) when parathyroidectomized dogs who developed tetany were given purified oxen (bovine) PTH with resolution of symptoms. He concluded that PTH plays an important role in maintaining normal blood calcium and phosphorus levels in the body. The full sequence of PTH was later described (2, 3).

Parathyroid glands are very small glands of the endocrine system, normally weighing approximately 25 g, and typically located to the side and behind the thyroid gland. Most individuals have four parathyroid glands, but rarely additional neck or ectopic parathyroid glands may be discovered during imaging or surgery. The role of these glands normally is to regulate calcium levels in the blood by release of PTH by sensing low serum calcium level through calcium-sensing receptors (CaSRs) located on the parathyroid cells. The serum calcium level is monitored and regulated tightly.

Parathyroid hormone regulates calcium homeostasis by a tightly controlled system. PTH plays an important role in mobilizing calcium from the skeleton where calcium is primarily stored and increasing calcium absorption from the intestine by increasing synthesis of calcitriol (1,25-dihydroxyvitamin D) from the kidney. Increased conversion of 25-hydroxyvitamin D to 1,25-dihydroxyvitamin D occurs in the proximal renal tubule. PTH also increases calcium reabsorption from the thick ascending limb of nephrons and facilitates the excretion of phosphorus through the kidneys.

Calcium is sensed by the CaSR, a 7-transmembrane G protein-coupled receptor found on the parathyroid glands, which stimulates PTH release in response to low serum calcium, and suppresses PTH release in response to high serum calcium. The CaSR is also expressed in several other tissues including renal tubular cells, where it regulates calcium reabsorption, as well as bone and intestinal cells. In hypercalcemia, the filtered calcium load overcomes the renal tubular ability to reabsorb calcium resulting in decrease in calcium and sodium transport in the loop of Henle with an associated decrease in urinary concentrating ability to reduce calcium absorption through the kidneys.

When the production of PTH is reduced or absent, low PTH is inadequate to maintain normocalcemia and normophosphatemia, thus the biochemical findings of the disease, such as hypocalcemia, hyperphosphatemia, and low PTH ensue. The most common cause of hypoparathyroidism is inadvertent damage to the parathyroid glands during thyroid surgery (4–6). Other causes of hypocalcemia that need to be ruled out include magnesium deficiency and vitamin D deficiency. Magnesium is needed for the secretion of PTH by the parathyroid glands and its depletion or excess may cause hypoparathyroidism and subsequent hypocalcemia. This is thought to be due to the lack or excess magnesium playing a role in defective cyclic AMP generation in the parathyroid glands interfering with PTH synthesis and secretion (7).

Symptoms of hypoparathyroidism are the result of low serum calcium effect on the internal organs and correlate strongly with the acuteness of the low serum calcium as well as the absolute level. For example, a patient who has chronic hypoparathyroidism since childhood may adapt to low levels of calcium even as low as 7.0 mg/dl and remain asymptomatic. Presenting symptoms are variable. Mild symptoms include numbness and tingling of the extremities and perioral region, muscle cramps, and fatigue, and in severe cases, tetany, seizure, altered mental status, cardiac rhythm disturbances, refractory congestive heart failure, bronchospasm, and laryngospasm can be seen (8, 9). In most patients, symptoms develop when the albumin-corrected serum calcium is less than 7.5–8.0 mg/dl (7).

## Prevalence

Epidemiologic studies estimate the incidence of hypoparathyroidism in the U.S. to 24–37/100,000 person-years, with an estimate of 60,000–80,000 affected individuals (10–12). Overall, 75% of cases are female and 25% male (13). About 75% of these patients are aged 45 years or older, and roughly 75% of cases are due to neck surgery and 25% are reported to be due to non-surgical causes. The most common cause of hypoparathyroidism is anterior neck surgery, i.e., secondary to thyroid or parathyroid or head or neck cancer surgery, which is reported to occur in 7–36% of surgeries, of which 38% are due to total thyroidectomy, 21% parathyroidectomy, 9% partial thyroidectomy, and 5% other neck surgeries (13). Most have transient hypoparathyroidism where parathyroid function recovers fully within in 6 months, and only 1–5% of patients develop permanent hypoparathyroidism lasting longer than 6 months (4, 13–16). Risk of hypoparathyroidism after anterior neck surgery depends on the experience of the surgeon. In addition, other factors that increase risk of hypoparathyroidism after neck surgery including, multigland parathyroid disease where more than one gland is removed, long-standing history of hyperparathyroidism resulting in hungry bone syndrome and in malabsorption resulting from weight loss surgeries [in particular roux-en-y gastric bypass surgery where absorption of calcium and vitamin D is affected (4)]. Vitamin D deficiency prior to surgery can result in postoperative hypocalcemia as well, and most experts recommend replacement of vitamin D to maintain vitamin D level in sufficient range (>20 ng/dl) prior to surgery. Vitamin D level should be individualized and corrected with caution in those individuals with primary hyperparathyroidism undergoing parathyroidectomy to prevent risk of hypercalcemia. The optimal 25-hydroxyvitamin D level is unknown in these individuals but some studies have shown a correlation between size of parathyroid adenoma and vitamin D deficiency (17).

Inherited causes of hypoparathyroidism are summarized in Table 1. Other rare, non-hereditary causes of hypoparathyroidism include parathyroid gland destruction such as irradiation and infiltrative disease (hemochromatosis, Wilson’s disease, granulomas, or metastatic cancer). In addition, neonatal hypocalcemia of prematurity or secondary to maternal hypercalcemia, hypo- or hypermagnesemia, and calcium sensor receptor antibodies. Pseudohypoparathyroidism (PTH resistance of kidney and bone presenting with high PTH and low calcium) is a separate disorder that can be seen rarely.

**Table 1:** **Inherited causes of hypoparathyroidism**.

Disease	Inheritance	Gene/protein	Chromosomal abnormalities	Associated findings
**Syndromic forms**				

Autoimmune polyglandular syndrome type 1 (APS-1)	Autoimmune recessive	AIRE gene mutation	21q22.3	Addison’s disease, pernicious anemia, diabetes mellitus type I, hypoparathyroidism, candidiasis, primary hypogonadism, autoimmune thyroid disease, alopecia, vitiligo

DiGeorge type I	Autosomal dominant	TBX1, NEBL	22q1.2/tbx1	Conotruncal abnormalities, abnormal facies, thymic aplasia, cleft palate, hypocalcemia, immunodeficiency, congenital heart defect, deformities of the ear, nose, and mouth

CHARGE	Autosomal dominant	CHD7, SEMA3E	8q12.1–q12.2,7q21.11	Coloboma of the eye, heart malformation, choanal atresia, retardation of growth and development, and genital and ear abnormalities, gonadotropin deficiency, anosmia

Hereditary deafness and renal dysplasia syndrome (HDR)	Autosomal dominant	GATA3	10p14	Deafness, renal dysplasia

Kenny–Caffey syndrome type I, Sanjad–Sakati syndrome	Autosomal dominant/recessive	TBCE	1q42.3	Short stature, osteosclerosis, cortical thickening of long bones, delayed anterior fontanel closure, basal ganglia calcification, hyperopia

Kenney–Caffey syndrome type 2	Autosomal recessive	FAM111A	11q12.1	

Dubowitz syndrome	Autosomal recessive	Unknown	?	Microcephaly, short stature, abnormal faces, and mild to severe mental retardation

Bartter syndrome type 5	Autosomal dominant	CaSR	3q21.1	May be accompanied by hypokalemia and metabolic alkalosis

Nephropathy, nerve deafness	Autosomal dominant	Unknown	?	

Nerve deafness without renal dysplasia	Autosomal dominant	Unknown	?	

Kearns–Sayre syndrome MELAS MTPDS	Maternal	Mitochondrial genome	?	Muscle defect: ophthalmoplegia, proximal muscle weakness, bilateral pigmented retinopathy, cardiac conduction abnormality, cerebral ataxia, deafness, diabetes mellitus, growth hormone deficiency, short stature

**Non-syndromic**				

Isolated hypoparathyroidism	Autosomal dominant	PTH, GCMB	11p15,6p24.2	
	Autosomal recessive X-linked	SOX3	Xq26–27	

ADH1	Autosomal dominant	CaSR	3q21.1	Hypocalcemia, hypercalcuria, normal or low PTH, low magnesium
ADH2	Autosomal dominant	GNA11	19p13	

*AIRE, autoimmune regulator 1; GCM2, glial cell missing 2; CaSR, calcium-sensing receptor; TBX1, T-box 1; NEBL, nebulette; CHD7, chromodomain helicase DNA-binding protein 7; SEMA3E, semaphorin 3E; GATA3, GATA-binding protein 3; TBCE, tubulin folding cofactor E; FAM111A, family with sequence similarity 111 member A; GCMB, glial cell missing gene; SOX3, Sry-related HMG box; GNA11, G protein subunit alpha 11; ADH, autosomal dominant hypocalcemia; MELAS, mitochondrial encephalopathy, stroke like episodes and lactic acidosis; MTPDS, mitochondrial trifunctional protein deficiency syndrome; ?, unknown*.

## Clinical Manifestations

Physical examination of a patient with hypocalcemia includes assessment of neuromuscular hyperexcitability by testing for Chvostek’s and Trousseau’s signs. Chvostek’s sign is elicited by tapping the facial nerve in front of the ear, causing the facial muscles on the ipsilateral side to twitch, causing contractions ranging from the upper lip and nose to the entire half-face when severe and significant hypocalcemia is present. Chvostek’s sign is positive in 15% of individuals with normal serum calcium. Trousseau’s sign is elicited by placing a blood pressure cuff around the arm and inflating the cuff to greater than the systolic pressure and holding this in place for 3 min, thereby occluding the brachial artery. The combination of absence of blood flow and hyperexcitability of the muscles due to hypocalcemia results in painful flexion of the wrist and metacarpophalangeal joints, with extension of the distal interphalangeal and proximal interphalangeal joints, and abduction of the fingers.

Clinical symptoms correlate with acuteness of hypocalcemia, as well as the absolute level of serum calcium. Patients with an acute drop in their serum calcium after neck surgery have more dramatic symptoms than those with chronic hypocalcemia. Myoclonic jerks, twitching, new-onset seizures due to cerebral hypocalcemia, or worsening of seizures are neurologic manifestations of hypocalcemia. Cardiac manifestations are prolonged QT interval and T-wave alternans, acute cardiomyopathy, and congestive heart failure due to decreased cardiac contractility related to low serum calcium and possibly PTH deficiency, as there are PTH receptors in cardiac myocytes.

Patients with hypoparathyroidism typically have higher bone density due to low bone turnover (6, 8, 18–20). Markers of bone turnover and iliac crest bone biopsy show a reduction in indices of bone turnover associated with an increase in trabecular and cortical width and density (21). Despite the higher bone density seen in these patients, the risk of fractures is not yet certain, although one study showed increased risk of upper extremity fracture in non-surgical hypoparathyroidism (11).

Impaired renal function is the most common complication seen in patients treated for hypoparathyroidism. It is associated with the age of the patient, duration of the disease, and level of hypercalcemia during treatment. Absence of PTH results in inability of renal tubules to reabsorb calcium, resulting in hypercalciuria and nephrocalcinosis. In addition, patients with underlying renal disease such as renal dysgenesis in DiGeorge syndrome are in particular at higher risk for nephrolithiasis. Overtreatment of hypoparathyroidism with calcitriol and calcium can lead to nephrolithiasis, thus monitoring of renal calcium excretion is necessary. One study found rates of chronic kidney disease Stage 3 or higher were 2- to 17-fold higher in patients with hypoparathyroidism than in normal individuals followed for 7 years (22). In addition, 50% of hypoparathyroid patients in this cohort had increased 24-h urine calcium and 25% had frank hypercalcuria. In patients who had renal imaging, 31% had nephrocalcinosis on CT scan or ultrasound of the kidneys, and 2–17% had renal impairment (5). Risk of hospitalization related to renal causes (kidney stones or chronic kidney disease) was higher in adult hypoparathyroid patients than normal controls (23). The same has been reported in children (24).

Many patients complain of cognitive dysfunction, in particular, brain fog, fatigue, and easy fatigability (9). Higher incidence of anxiety, depression, and overall reduced quality of life occurs in patients with hypoparathyroidism compared to normal control groups (8, 25, 26). However, one systematic review failed to show a link between PTH and cognitive dysfunction and dementia (27), so it is assumed that these conditions are mostly due to the calcium disturbance.

Recent studies have shown an increased risk of posterior subcapsular cataracts, likely due to elevated calcium × phosphorus product occurring in lenses in the eyes. Basal ganglia and intracerebral calcifications may be associated with extrapyramidal movement disorders and psychosis, but not all studies have confirmed an association. Increased risks of cardiac arrhythmias and cardiovascular diseases have also been reported (11, 22, 23). Other studies have suggested an increased incidence of infection in these individuals, although the mechanism is not clear (11).

## Biochemical Features

Albumin-corrected serum calcium and 1,25(OH)_2_D are typically low, with an increased serum phosphorus level. Intact PTH is typically low but can be inappropriately normal for the degree of hypocalcemia. Twenty-four-hour urine calcium is most often increased, but may be normal or low before calcium supplementation is started, depending on calcium intake and bone turnover (28). Measurement of 25-hydroxyvitamin D, magnesium, and kidney function are critical in helping to understand the physiology of individuals affected by hypoparathyroidism.

## Physical Examination

Physical examination includes an examination of the neck for scars that might suggest postsurgical hypoparathyroidism, evaluation for mucocutaneous candidiasis, or vitiligo that may suggest autoimmune polyglandular syndrome type 1, and generalized bronzing and signs of liver disease that may suggest hemochromatosis or other causes of iron overload. In addition, growth retardation, congenital anomalies, hearing loss, or mental retardation suggest the possibility of genetic syndromic causes of the disease.

## Treatment

### Conventional Therapy

Careful surgical planning in patients with hyperparathyroidism with preoperative localization of the parathyroid tumor may reduce the likelihood of postoperative parathyroid gland dysfunction. If an extensive surgical procedure is undertaken, preservation with reimplantation of parathyroid tissue should be done. Presurgical optimization of vitamin D is helpful, given that vitamin D deficiency is very common in the older population and patients undergoing parathyroid surgery. Caution with vitamin D replacement prior to surgery is appropriate due to concerns that too much vitamin D could worsen hypercalcemia. Studies have shown that vitamin D supplementation improves bone health in these individuals (4).

To date, three guidelines on the management of hypoparathyroidism have been published, including those of the International Consensus Conference guidelines based on the first international conference on hypoparathyroidism held in Florence, Italy, in May 2015, the European Society of Endocrinology clinical guidelines, and the American Association of Clinical Endocrinologists and American College of Endocrinology Disease State Clinic Review (4, 16, 29). In addition, three papers were published in 2016 on presentation, epidemiology, diagnosis, and management of hypoparathyroidism (6, 12, 30). These guidelines address management of acute and chronic hypoparathyroidism. Long-term management of hypoparathyroidism should target goal serum calcium within the low-normal range, with serum phosphorus within the high-normal range, and avoid significant hypo- or hypercalcemia. The goal is to reduce symptoms, minimize risk of kidney stones and kidney dysfunction, and prevent ectopic soft tissue calcium deposition. With low or absent PTH, calcium absorption is dependent on daily calcium and active vitamin D intake, so patients with hypoparathyroidism require daily consistent calcium and active vitamin D intake necessary to achieve goal serum calcium. Intake of other medications such as iron can interfere with calcium absorption.

The goal of therapy is to maintain serum calcium level at 8.0–9.0 mg/dl range while normalizing urinary calcium. Standard treatment consists of adequate calcium, vitamin D2 or D3, active vitamin D (1,25-dihydroxyvitamin D3), and magnesium supplementation as needed (Table 2). High doses of vitamin D2 or Vitamin D3 supplementation may increase action of vitamin D and vitamin D may also have extraskeletal effects in some tissues. Calcium carbonate (containing 40% elemental calcium) and citrate (containing 21% elemental calcium) are the most commonly prescribed preparations given, typically in divided doses two to four times per day. For patients who have hypo- or achlorhydria due to use of antacids or proton pump inhibitor therapy, or elderly patients with low gastric acid secretion, calcium citrate is the preferred preparation for the management of hypocalcemia because calcium carbonate requires an acidic gastric environment for digestion and absorption, whereas calcium citrate is easily absorbed without stomach acid. Calcium carbonate should be taken with food, unlike calcium citrate, which does not require food intake to absorb. Magnesium supplementation may be necessary if serum magnesium is low, as normal serum magnesium is required for normal secretion of PTH (31). Twenty-four-hour urine calcium and creatinine should be monitored every 6 months to once a year to monitor for hypercalcuria. Thiazide-type diuretics (hydrochlorothiazide, chlorthalidone, and indapamide) and sodium restriction may be added to reduce urine calcium if hypercalcuria is present.

**Table 2:** **Calcium and vitamin D metabolites in the management of chronic hypoparathyroidism**.

Medication	Typical dose
Calcium carbonate	1,000–9,000 mg elemental calcium/day in 2–4 divided doses
Calcium citrate	1,000–9,000 mg elemental calcium/day in 2–4 divided doses
**Vitamin D preparation**	
Ergocalciferol (D_2_) or cholecalciferol (D_3_)	Total 25-hydroxyvitamin D level ≥30 ng/ml
Calcitriol [1,25(OH)_2_D_3_]	0.25–2.0 μg/day
Alfacalcidol (1-alphaOH-vitamin D_3_)	0.5–4.0 μg/day (not available in U.S.)
**Thiazide diuretics/other**	
Hydrochlorothiazide	12.5–100 mg/day
Chlorthalidone	25–100 mg/day longer duration
Indapamide	1.25–5 mg/day
Amiloride	5 mg daily (alone or combined with hydrochlorothiazide)

Despite standard therapy, some individuals remain symptomatic or fail to meet treatment goals, with some requiring multiple hospitalizations for the treatment of hypocalcemia or complications of hypocalcemia. For those patients with difficult to control hypoparathyroidism, new adjunctive therapy options are available (29, 30).

## Emerging Treatments

### PTH 1-34

Teriparatide (PTH 1-34) is a truncated molecule of synthetic PTH approved for management of osteoporosis, but not hypoparathyroidism. PTH 1-34 has been evaluated in a number of studies in adult and pediatric patients with postsurgical hypoparathyroidism (32) and permanent hypoparathyroidism from other causes (33–38) and appears to be safe and efficacious. Urinary calcium in PTH 1-34 treated patients was decreased when injections were given once a day compared to calcitriol-treated controls (35, 37). In a study of 14 children treated with twice a day PTH 1-34 for 28 weeks vs. once a day injection, serum calcium, serum magnesium, and urine calcium parameters were better controlled in the twice daily injection group compared to the once daily treatment group, especially in the second half of the day (12–24 h) (35). When PTH 1-34 was given by an insulin pump as a continuous infusion, urine calcium was reduced by 50%, and serum calcium was near normal, with minimal fluctuation compared to twice daily injections (38). Improvement in mental and physical health has been reported with PTH 1-34 treatment (39).

### Recombinant Human Parathyroid Hormone (rhPTH) 1-84

The full-length form of rhPTH (1-84; Natpara) was the first approved form of PTH approved by the FDA in January 2015 for adjunctive therapy in management of hypoparathyroidism in individuals not adequately controlled with calcium, active vitamin D, thiazide-type diuretics, and/or magnesium therapy as needed. Natpara is given as a once a day subcutaneous injection based on phase 1 clinical trial data in adults with hypoparathyroidism showing that single daily injections caused improvement in serum calcium for 24 h (40). rhPTH 1-84 initially was evaluated for management of osteoporosis and approved in Europe in 2006, but due to increased risk of hypercalcemia in the U.S. trial, the FDA declined to approve it.

An open-label trial of 27 patients treated with rhPTH 1-84 every other day at 100 μg/dose for 4 years showed that all patients had stable serum calcium and were able to reduce their calcium and active vitamin D supplementation significantly (41). Twenty-four-hour urine calcium was also reduced at 4 years with treatment, and serum phosphorus declined (41). Six-year data from this cohort was recently published, extending these findings (42). The long-term efficacy of PTH replacement therapy on kidney function preservation is not yet known.

REPLACE was, the pivotal randomized, double-blind, placebo-controlled trial for 24 weeks conducted with 134 adults with hypoparathyroidism randomized 1:2 to placebo or rhPTH 1-84. Subjects were injected once daily with rhPTH 1-84 vs. placebo with dose titration from 50 to 75 or 100 μg/day (5). The trial showed that 53% of treated subjects were able to reduce their supplemental calcium and active vitamin D doses by ≥50%, with 43% able to completely stop all active vitamin D and reduce their calcium dose to 500 mg/day or less, while maintaining normal serum calcium.

Markers of bone turnover are typically reduced, reflecting low bone turnover, along with higher bone density due to increased trabecular and cortical bone volume, in individuals with untreated hypoparathyroidism, but it is not yet clear whether this denser bone translates into increased bone strength (18, 41). Treatment with rhPTH 1-84 increased lumbar spine BMD, but did not change femoral neck or total hip BMD, whereas the one-third distal radius BMD declined at 2 years but remained stable at 4 years (41). Paired transiliac bone biopsies before and after treatment with rhPTH (1-84) showed that 1 year treatment resulted in reduction in bone mineralization and high bone turnover, but these parameters returned to baseline at 2 years (43, 44).

At baseline, hypoparathyroidism patients have low to low-normal bone turnover markers, and with treatment with rhPTH (1-84), there was a threefold increase in markers of bone resorption [e.g., tartrate-resistant acid phosphatase 5b and collagen type 1 crosslinked C-telopeptide (CTx-telopeptide)], and bone formation markers (e.g., bone specific alkaline phosphatase, osteocalcin, and total procollagen type 1 N-terminal propeptide), peaking at 6–12 months, with a slow decline to steady state by 30 months, but remaining higher than pretreatment values (41).

Quality of life among patients treated with rhPTH (1-84) 100 μg every other day for 5 years assessed by the SF-36 showed an improvement compared to untreated individuals (45). On the contrary, there was no change in muscle strength, postural stability or quality of life at 6 months in the phase III randomized controlled trial of hypoparathyroid patients receiving rhPTH (1-84) (46).

### Safety

Long-term data on the safety and efficacy of PTH in any form beyond 6 years is not available. In the REPLACE trial, a single subject taking PTH (1-84) 100 μg each day had hypercalcemia, which required dose reduction to 50 μg each day, and did not require discontinuation of treatment. Muscle spasm, hypocalcemia, paresthesia, headache, and nausea were the most common adverse events reported in those receiving rhPTH (1-84) compared to placebo, most of which occurred during dose titration at the beginning of the study (5). Cusano et al. reported 11 episodes of mild hypercalcemia in eight subjects over 4 years of treatment with PTH (1-84) 100 μg every other day, most of which occurred within the first 6 months of treatment during dose titration, and corrected with adjustment of calcium and vitamin D intake (41).

Longer term studies are needed to address the potential safety concerns related to osteosarcoma seen in the Fisher 344 strain of rat treated with rhPTH 1-34 in preclinical studies, which was dose and treatment duration dependent (47). The U.S. post-marketing surveillance study of adults treated with teriparatide for over 7 years did not report an association between rhPTH 1-34 treatment and osteosarcoma in humans (48).

Long-term effects of Natpara are unknown. Given the lack of such data, it is recommended that rhPTH 1-84 be prescribed only for those patients who cannot be well controlled on calcium and active forms of vitamin D, and for whom the potential benefits are considered to outweigh the potential risks.

NPS Pharmaceuticals, Inc./Shire Pharmaceuticals has designated a Natpara REMS registry to monitor potential adverse effects of Natpara. Similar to rhPTH 1-34, Natpara has a black box warning to avoid use in those individuals with increased baseline risk of osteosarcoma including those with Paget’s disease of bone, unexplained elevation of serum calcium or alkaline phosphatase, pediatric and young adult patients with open epiphyses, patients with hereditary disorders predisposing to osteosarcoma, or patients with a history of prior external beam or implant radiation therapy involving the skeleton.

## Future Directions

Future therapeutic options include additional PTH analogs with different kinetics, administration of PTH *via* pump or implantable devices, parathyroid gland transplantation, or ultimately regeneration of parathyroid glands by stem-cell therapy.

## Conclusion

Hypoparathyroidism is no longer a rare disease without treatment options. rhPTH 1-84 (Natpara) appears to be a promising treatment option for those individuals who are not well controlled on current standard therapy with calcium, active vitamin D, thiazide-type diuretics, and/or magnesium as needed. Additional studies are needed to evaluate if Natpara provides benefit in improving comorbidities and complications of hypoparathyroidism, including quality of life, neuropsychiatric conditions, kidney dysfunction, kidney stones, extraskeletal calcifications, cataracts, and skeletal strength and fracture.

## Author Contributions

Both authors contributed equally to the manuscript preparation.

## Conflict of Interest Statement

EA has no conflict of interest to disclose. BC received research funding from NPS Pharmaceuticals, Inc./Shire.
